# Efficacy and safety of thunder-fire moxibustion for patients with knee osteoarthritis: A protocol for systematic review and meta-analysis

**DOI:** 10.1097/MD.0000000000025384

**Published:** 2021-04-09

**Authors:** Qiaotong Huang, Jun Chen, Yunfeng Jiang, Lunbin Lu, Siyuan Zhu, Zhiying Zhong, Genhua Tang, Xingchen Zhou, Han Guo

**Affiliations:** a The Affiliated Hospital of Guangxi University of Traditional Chinese Medicine, Nanning; b Jiangxi University of Traditional Chinese Medicine, Nanchang, China.

**Keywords:** knee osteoarthritis, meta-analysis, protocol, systematic review, thunder-fire moxibustion

## Abstract

**Background:**

Knee osteoarthritis (KOA) is a major public health issue because it causes pain and functional limitation in patients. Many studies have reported that moxibustion, a treatment in traditional Chinese medicine, is effective in treating KOA. The aim of this protocol is to develop a standard in advance for synthesize and assess the efficacy and safety of thunder-fire moxibustion for KOA from these randomized controlled trial.

**Methods:**

The 2 commentators will screen 7 databases (PubMed, EMBASE, the Cochrane Library, Chinese National Knowledge Infrastructure, Chinese VIP Information, Wanfang Database, and Chinese Biomedical Literature Database) for randomized controlled trials that can be included from the time the database is built up until publication in December 2020. The original study that randomized control trials of thunder-fire moxibustion for patients with KOA will be selected and is not limited by country or language. In addition, researches in progress, the reference lists, and the citation lists of identified publications will be retrieved similarly. Study selection, data extraction, and assessment of the quality will be performed independently by 2 reviewers who have been trained before data extraction. A meta-analysis will be conduct if the quantity and quality of the original studies included are satisfactory; otherwise, a descriptive analysis will be conducted. Review Manager 5.4 software (The Nordic Cochrane Centre, The Cochrane Collaboration, Copenhagen, Denmark) will be using for data synthesis and assessment the risk of bias according to Cochrane Handbook.

**Result:**

This study will provide a comprehensive review of current evidence for the treatment of thunder-fire moxibustion on KOA.

**Conclusion:**

The conclusion of this study will provide a judging basis that whether the treatment of KOA with thunder-fire moxibustion is effective.

**Registration number:**

INPLASY2020100012.

## 1 Introduction

Knee osteoarthritis (KOA) is a disease with cartilage and bone joint destruction as the main pathological manifestations. It is very common, progresses slowly, and cannot be completely cured, which causes significant discomfort, pain, and disability.^[1]^ As a common site of osteoarthritis, KOA follows the course of osteoarthritis. All structures in the joint, including hyaline articular cartilage, capsule, and muscles around the joint, will change in nature and function. In some patients, synovitis, ligamentous laxity, and bone marrow lesions may be a sign of bone injury.^[2]^ The knee joint consists of the following 3 joint chambers: the lateral tibiofemoral compartment, the medial tibiofemoral compartment, and the patellofemoral compartment. The most common site of arthritis is the patella femoral joint.^[3]^

As the 11th highest contributor to global disability. The prevalence of KOA increases with age and is more common in women than men. The global age-standardized prevalence of knee OA was 3.8% (95% uncertainty interval 3.6%–4.1%).^[4,5]^

Magnetic resonance imaging findings of osteoarthritis, including meniscal tears, are common in middle-aged and older adults with and without knee pain.^[6]^ Radiographs findings in patients with osteoarthritis are poorly correlated with pain severity, It is even possible that the patient's radiographs are normal.^[7]^

The etiology and exact pathogenesis (biomechanical, biochemical, or otherwise) of KOA are still not fully understood, and there is no cure for KOA.^[8]^ At present, the common treatment methods include: drug therapy, surgery treatment, traditional Chinese medicine treatment, exercise therapy, etc, and there are many targeted characteristic therapies like stem cells,^[9]^ platelet rich plasma,^[10]^ and cytokine modulation are popularizing to patients. The mother of all interventions is to alleviating pain, attempting to rectify mechanical malalignment, and identifying and addressing manifestations of joint instability.^[11]^ Common drug treatments include: nonsteroidal anti-inflammatory drugs, cyclooxygenase-2 inhibitors, and acetaminophen present. Nonsteroidal anti-inflammatory drugs,^[12]^ as the first-line drug, has the remarkable performance of alleviating pain and alleviating symptoms. However, it also has some adverse reactions, such as common toxic reactions of drugs, drug dependence, and drug resistance.^[13,14]^ Surgical treatment includes joint replacement, joint puncture, and occlusion. For most patients, joint replacement books are faced with problems such as high price and large adverse reactions. Therefore, intra-articular injection of hyaluronic acid is one of the more accepted treatment methods. Although some studies have shown that injection of hyaluronic acid and intra-articular corticosteroid injections can improve knee pain,^[15–17]^ some efficacy evaluations have suggested that the efficacy of injection of hyaluronic acid may be overestimated.^[11]^ Exercise therapy plays a very important role as a daily treatment for patients with mild disease and patients in remission, or as a combined treatment for patients with severe disease.

In addition to conventional modern medicine medication, the effect of traditional Chinese medicine treatment can not be ignored, such as traditional Chinese medicine treatment for the alleviation of patients’ symptoms has a certain effect, acupuncture, and moxibustion as a part of traditional medicine, electro-acupuncture, moxibustion therapy has become a popular treatment for KOA and is associated with a low risk of adverse reaction.^[18,19]^

In recent years, more and more studies tend to evaluate the efficacy and safety of various therapies in the treatment of KOA in order to find better treatment options.

Acupuncture is an effective traditional therapy in China. Thunder-fire moxibustion^[20]^ is one of the traditional operation methods of acupuncture. Through the strong stimulation of the acupoints, in order to dredge the meridians, accelerate the flow of Qi and blood, so that pain relief. Thunder-fire moxibustion, as a nondrug treatment, has the advantages of low adverse reaction rate and small harm.^[21]^ Therefore, the purpose of this study is to summarize the original studies on the treatment of KOA with thunder and fire moxibustion, in order to evaluate whether thunder and fire moxibustion is effective in the treatment of KOA.

## 2 Methods

### 2.1 Registration

This protocol will be reported according to the preferred reporting items for systematic reviews and meta-analyses protocols.^[22]^ It is registered in the INPLASY (registration number, INPLASY2020100012; https://inplasy.com/inplasy-2020-10-0012/).

### 2.2 Inclusion criteria for this overview

PICOS will be applied, including population, intervention, comparison, outcome, and study.

#### 2.2.1 Types of studies

Randomized controlled trials (RCTs) with thunder-fire moxibustion as the primary intervention for KOA will be included, and other studies such as case reports and reviews will be excluded. No restrictions on country but language will be limited on English and Chinese.

#### 2.2.2 Types of participants

Participants with KOA manifestations^[23]^ (persistent knee pain, limited morning stiffness, reduce function, crepitus, restricted movement, and bony enlargement) will be included after a detailed clinical diagnosis. Not subject to gender, age, race, and other restrictions.

#### 2.2.3 Types of interventions

Without limits on course and dose, we will include studies in which thunder-fire moxibustion is the primary intervention and, if necessary, we will include studies in which thunder-fire moxibustion is combined with other active treatments versus active treatment alone.

#### 2.2.4 Types of comparisons

The selected RCTs should testify that the interventions were compared with a control group composed of placebo, sham acupuncture, no treatment or other active therapies.

#### 2.2.5 Outcomes

Primary outcome: VAS scores.

Secondary outcomes: WOMAC, KOOS, body composition analysis, knee range of motion test.

### 2.3 Search methods for study identification

#### 2.3.1 Electronic searches

Two investigators will retrieve the relevant RCTs in the following databases: PubMed, Embase, the Cochrane Library, Chinese National Knowledge Infrastructure, Chinese VIP information, Wanfang Database, and Chinese Biomedical Literature Database, from inception until December 2020 without restriction to publication status but limited with languages. A comprehensive search strategy will be conducted, various combinations of MeSH items and free words will be searched synchronously, including “knee osteoarthritis,” “thunder-fire moxibustion,” “lei huo jiu,” etc. The preliminary search strategy for PubMed is presented in Table 1.

**Table 1:** Search strategy (PubMed).

Order	Strategy
#1	Search “Osteoarthritis, Knee”[Mesh] Sort by: Publication Date
#2	Search (((Knee Osteoarthritis[Title/Abstract]) OR Knee Osteoarthritides [Title/Abstract]) OR Osteoarthritis of Knee [Title/Abstract]) Sort by: Publication Date
#3	#1 OR #2
#4	Search ((((randomized controlled trial[Publication Type]) OR controlled clinical trial[Publication Type]) OR randomized[Title/Abstract]) OR randomly [Title/Abstract]) OR trial[Title/Abstract]) Sort by: Publication Date
#5	Search (humans[MeSH Terms]) NOT animals[MeSH Terms] Sort by: Publication Date
#6	#4 AND #5
#7	Search (Acupuncture [Mesh] OR Moxibustion [Title/Abstract]) Sort by: Publication Date
#8	Search (thunder-fire moxibustion [Title/Abstract]) OR “lei huo jiu” [Title/Abstract]) Sort by: Publication Date
#9	#7 OR #8
#10	#3 AND #6 AND #9

#### 2.3.2 Searching other resources

The relevant published references and citation list will be retrieved in Web of Science. In addition, the relevant systematic reviews or overview will also be identified for additional relevant studies. Moreover, relevant paper versions of medical journals and journals will be screened to ensure that the original studies that not included in the electronic databases could be included possibly.

### 2.4 Data collection and analysis

#### 2.4.1 Study selection

**Figure 1:**
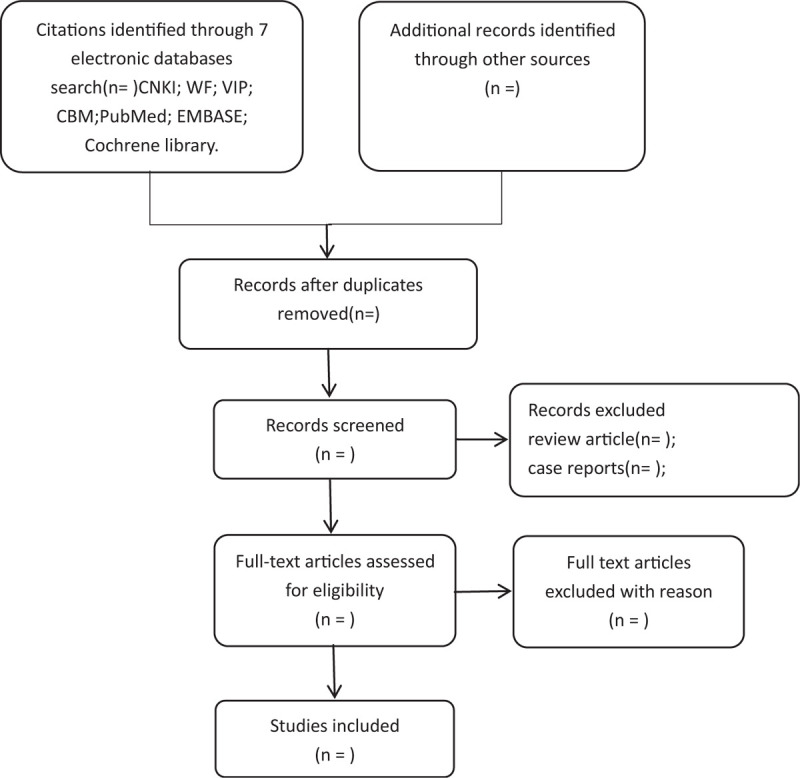
Flowchart of literature selection.

All reviewers undergo rigorous training before selecting the study. Preliminary screening of the study will be conducted by 2 reviewers independently. After searching, the duplicated studies will be removal initially from the retrieved studies by Endnote(X9). And then, 2 independent reviewers (QT H and LB L) will screened titles, abstracts, and keywords of all retrieved studies for candidates according to the inclusion and exclusion criteria, we will obtain the full text of all possibly relevant studies. Excluded studies will be recorded with explanations. If it is uncertain whether to adopt because of the lack of information, LB L will try to contact authors of the original reports to obtain the information of lost. During the procedure, disagreements will be resolved by discussion or consensus with the third reviewer (YF J). Study selection will be performed in accordance with the preferred reporting items for systematic reviews and meta-analyses flowchart (Fig. 1).

#### 2.4.2 Data extraction and management

A unified data extraction table will be designed before data extraction, and data extraction will also be carried out independently by 2 reviewers (SY Z and GH T). The proposed extracted information includes:

(1) General information: author, country, year of publication, study design, and database;
(2) Population characteristics: sex, age, baseline diseases, and sample size;
(3) Methodological characteristics: information sources, intervention(s), comparison(s), bias assessment, etc.

Any objections will be discussed by 2 reviewers, and further objections will be arbitrated by the third author (ZY Z).

#### 2.4.3 Assessment of risk of bias

To systematically evaluate the quality of each of the studies that final included. Two reviewers (SY Z and LB L) will assess the risk of bias for each included study according to the Cochrane handbook. It will eventually be rated on 3 levels (“ high risk of bias,” “medium risk of bias,” and “low risk of bias”).^[12]^ The specific evaluation items include the following 7 aspects: generation of random sequence, allocation concealment, blindness of participants and personnel, blindness of outcome assessment, incomplete outcome data, selective reporting, and other bias.

#### 2.4.4 Measures of treatment effect

Review Manager 5.4 (The Nordic Cochrane Centre, The Cochrane Collaboration, Copenhagen, Denmark) will be used for data analysis and quantitative data synthesis. We will use the weight mean difference and 95% confidence interval to measure the continuous variables, while the results of dichotomous variables will using risk ratio and its 95% confidence interval.

#### 2.4.5 Dealing with missing data

If the specific information we need to collect are not be reported, the reviewer (GH T) will attempt to contact the original author for relevant information by telephone or e-mail. If the required information is not available, it will be explained in the article. Then, the missing data will be assumed to be “missing at random” and “missing not at random” according to the Cochrane Handbook.^[13]^ For the data missing at random, the analysis will rely on existing data, while we will filling the missing data with replacement values and make a sensitivity analysis to examine the potential impact of missing information, if necessary.

#### 2.4.6 Assessment of heterogeneity

Heterogeneity refers to the difference between studies in the systematic review ^[14]^ and the value of *I*^2^ represents the heterogeneity after data synthesis. We will use *I*^2^ to assess statistical heterogeneity between trials. If the *I*^2^ < 50%, that indicates slight or no heterogeneity in the evidence of the combined results, while *I*^2^ ≥ 50%, it means studies with high heterogeneity. The fixed effects model will be adopted when the *P* > .1 and *I*^2^ < 50%, while apply the random effect if *P* < .1 and *I*^2^ ≥ 50%.

#### 2.4.7 Assessment of reporting bias

An assessment of the reported bias will be presented in the form of a funnel plot. If the points on both sides of the funnel plot are scattered and asymmetric, it is considered that there is a report bias and the reliability of this study is low. On the contrary, if the point distribution on both sides of the funnel plot is symmetrical, we believe that there is no or very low reporting bias, and the results of this study are reliable.

#### 2.4.8 Data synthesis and subgroup analysis

All analysis will be done through RevMan 5.4. According to heterogeneity assessment, mean difference or risk ratio were calculated using fixed or random effects models. In addition, if the *I*^2^ obtained after data consolidation is greater than 50% and the *P*-value is less than .1, sensitivity or subgroup analysis will be performed to exclude the source of heterogeneity. If the included original research data is insufficient for quantitative analysis, the review will only represent and summarize the evidence.

#### 2.4.9 Sensitivity analysis

If the results show significant heterogeneity and the number of included studies is sufficient, sensitivity analysis will be performed to identify the quality and robustness of the meta-analysis result, which includes assessing the impact of sample size, methodological elements, and the characteristic of research and missing data.

#### 2.4.10 Grading the quality of evidence

The quality of evidence will be evaluated using the grading of recommendations assessment, development, and evaluation.^[15]^ The quality of evidences will be rated on 4 levels (high, medium, low, or very low). Two reviewers (XC Z and HG) will conduct the assessment process separately and describe in detail the reasons for downgraded or upgraded outcomes affecting the quality of evidence to guarantee the reliability and transparency of results.

## 3 Discussion

Due to the long course of disease, serious and irreversible pathological changes, the disability rate of Knee OA ranks 11th in the world, which has a great impact on the lives of patients and a great burden on social medical care. Because the pathogenesis is not clear, according to the current treatment principles, drug therapy like nonsteroidal anti-inflammatory drugs and cyclooxygenase-2 inhibitors is still the main treatment measures, including, but drug therapy is often associated with certain side effects. At the same time, sports therapy and acupuncture and moxibustion in traditional medicine are often used as complementary and alternative therapies.

However, due to the lack of evidence-based evidence, the efficacy and safety of thunder-fire moxibustion in the treatment of OA cannot be guaranteed, and some studies have reported that acupuncture may be a placebo effect. So far, there has not been a comprehensive review of the efficacy of thunder-fire moxibustion.

The purpose of this study is to evaluate the efficacy of thunder-fire moxibustion in the treatment of KOA and to provide a reliable treatment plan for clinical staff. In addition, through this study, it is believed that more and higher quality original research will be conducted to provide more accurate information.

## Acknowledgments

The authors would like to thank the following people who either provided feedback on the protocol or supported the development of the methods: Qiaotong Huang^1^, Jun Chen^2^, Yunfeng Jiang^3^, Lunbin Lu^4^, Siyuan Zhu^5^, Zhiying Zhong^6^, Genhua Tang^7^, Xingchen Zhou^8^, Han Guo^9^.

Authors’ information

^1^ The Affiliated Hospital of Guangxi University of Traditional Chinese Medicine, Nanning, China

^2^ Jiangxi University of Traditional Chinese Medicine, Nanchang, Jiangxi province, China.

^3^ Jiangxi University of Traditional Chinese Medicine, Nanchang, Jiangxi province, China.

^4^ Department of Acupuncture and Moxibustion, Graduate College, Jiangxi University of Traditional Chinese Medicine, Nanchang, Jiangxi province, China.

^5^ Department of Acupuncture and Moxibustion, Graduate College, Jiangxi University of Traditional Chinese Medicine, Nanchang, Jiangxi province, China.

^6^ Department of Acupuncture and Moxibustion, Graduate College, Jiangxi University of Traditional Chinese Medicine, Nanchang, Jiangxi province, China.

^7^ Department of Acupuncture and Moxibustion, Graduate College, Jiangxi University of Traditional Chinese Medicine, Nanchang, Jiangxi province, China.

^8^ Department of acupuncture and Moxibustion, The Affiliated Hospital of Jiangxi University of Traditional Chinese Medicine, Nanchang, China.

^9^ Department of acupuncture and Moxibustion, The Affiliated Hospital of Jiangxi University of Traditional Chinese Medicine, Nanchang, China.

## Author contributions

**Conceptualization:** Qiaotong Huang, Jun Chen.

**Data curation:** Jun Chen, Yunfeng Jiang.

**Formal analysis:** Qiaotong Huang, Lunbin Lu.

**Investigation:** Siyuan Zhu, Zhiying Zhong, Genhua Tang.

**Methodology:** Lunbin Lu, Xingchen Zhou, Han Guo.

**Software:** Qiaotong Huang, Yunfeng Jiang, Lunbin Lu.

**Writing – original draft:** Qiaotong Huang, Lunbin Lu, Siyuan Zhu, Zhiying Zhong, Genhua Tang.

**Writing – review & editing:** Jun Chen, Yunfeng Jiang, Xingchen Zhou.
